# Feeding Experience of *Bemisia tabaci* (Hemiptera: Aleyrodidae) Affects Their Performance on Different Host Plants

**DOI:** 10.1371/journal.pone.0077368

**Published:** 2013-10-11

**Authors:** M. Mostafizur Rahman Shah, Tong-Xian Liu

**Affiliations:** 1 State Key Laboratory of Crop Stress Biology in Arid Areas, Northwest A&F University, Yangling, Shaanxi, China; 2 Key Laboratory of Integrated Pest Management on the Loess Plateau of Ministry of Agriculture, Northwest A&F University, Yangling, Shaanxi, China; 3 Wheat Research Center, Bangladesh Agricultural Research Institute, Dinajpur, Bangladesh; French National Institute for Agricultural Research (INRA), France

## Abstract

The sweetpotato whitefly, *Bemisia tabaci* biotype B is extremely polyphagous with >600 species of host plants. We hypothesized that previous experience of the whitefly on a given host plant affects their host selection and performance on the plants without previous experience. We investigated the host selection for feeding and oviposition of adults and development and survival of immatures of three host-plant-experienced populations of *B. tabaci*, namely Bemisia-eggplant, Bemisia-tomato and Bemisia-cucumber, on their experienced host plant and each of the three other plant species (eggplant, tomato, cucumber and pepper) without previous experience. We found that the influence of previous experience of the whiteflies varied among the populations. All populations refused pepper for feeding and oviposition, whereas the Bemisia-cucumber and the Bemisia-eggplant strongly preferred cucumber. Bemisia-tomato did not show strong preference to any of the three host palnts. Development time from egg to adult eclosion varied among the populations, being shortest on eggplant, longest on pepper, and intermediate on tomato and cucumber except for the Bemisia-cucumber developed similarly on tomato and pepper. The survivorship from egg to adult eclosion of all populations was highest on eggplant (80-98%), lowest on pepper (0-20%), and intermediate on tomato and cucumber. In conclusion, the effects of previous experience of whiteflies on host selection for feeding and oviposition, development, and survivorship varied depending on host plants, and host plants play a stronger role than previous experience. Preference of feeding and oviposition by adults may not accurately reflect host suitability of immatures. These results provided important information for understanding whitefly population dynamics and dispersal among different crop systems.

## Introduction

The sweetpotato whitefly, *Bemisia tabaci* Gennadius (=*Bemisia argentifolli* Bellows and Perring) (Hemiptera: Aleyrodidae) biotype B, is one of the most important pest insects of horticultural and some of agronomic crops in the tropics and subtropics of the world and in confined surroundings of other areas [1,2]. In 1949, *B. tabaci* was first documented in China [3], though it was not considered a pest until the mid 1990s [4,5]. In 1990s, it became a destructive pest in China that was recognized as an invasive new biotype, biotype ‘B’ [6-8]. Subsequently, the whitefly has been recorded injuring crops and ornamentals in most provinces in China and economic losses caused by this pest continuously increased [9]. It has been recorded from a total of 361 plant species in 89 families in south, southeast, middle, north and northwest of China where many vegetables, filed crops and ornamental crops in the families of Compositae, Cruciferae, Cucurbitaceae, Solanaceae and Leguminosae were the most preferred hosts [8,10-12].

*Bemisia tabaci* shows dissimilar preference concerning their host suitability, oviposition, adaptation, and efficiency of virus transmission [7,13]. It has a diverse host range [14,15] and consequently easily adapts to new hosts and geographic regions. Apart from Antarctica, it has been recorded throughout the world [7,16], while it infests almost 600 plant host species, including eggplant, tomato, cucumber, and pepper [17,18]. These plant hosts could change the fitness of establishment of *B. tabaci* biotype B putative species [19,20].

*Bemisia tabaci* exhibited significant oviposition preference among plant hosts [21] and leaf surfaces of the same host [21,22]. Their oviposition preference is positively correlated with host quality for their offspring performance [23-25]. Nonetheless, prior feeding experience [26] and diverse plant distribution [27,28] might mediate the performance of phytophagous insects.

Although a large number of investigations have examined the performance of putative species of *Bemisia tabaci* biotype B on various plant hosts [15,29-35], few studies have compared the differences in fitness of *B. tabaci* populations with previous feeding experience on particular host species. We hypothesized that previous experience of the whitefly on a given host plant affects their host selection and performance on plants compared to populations without previous experience on that plant. We tested our hypothesis using three host-plant-experienced populations of *B. tabaci*, namely Bemisia-eggplant, Bemisia-tomato and Bemisia-cucumber, and determined host selection for feeding and oviposition of adults, and development and survival of immatures on their natal host plant and each of the three other plant species (tomato, eggplant, cucumber and pepper). 

## Materials and Methods

The study described was carried out in the Key Laboratory of Applied Entomology, Northwest A&F University, Yangling, Shaanxi, China from October 2011 to January 2013.

### Host Plants

Eggplant, *Solanum melongena* L. (Solanaceae) cv. ‘Zichangqie’; tomato, *Solanum lycopersicum* L. (Solanaceae) cv. ‘Florida Lanai’; cucumber, *Cucumis sativus* L. (Cucurbitaceae) cv. ‘Jinchun’; and pepper, *Capsicum annuum* L. (Solanaceae) cv. ‘Qiemen-Tianjiao’ were used. Plant seeds were germinated and seedlings were raised in plastic potting trays with a potting mix (a mixer of peat moss, vermiculite, and perlite at 5:1:1 ratio by volume) inside a growth chamber maintaining at 25±1°C, 75±5% RH, and a photoperiod of 16L:8D h at a light intensity of 1400-1725 lux. The plant seedlings were individually transplanted in 15 diameter plastic pots with the same potting mix for mass-rearing of whiteflies. The plants with whiteflies were maintained inside air-conditioned insectaries at 25 ± 2°C, 65 ± 5% RH, and a photoperiod of 16L:8D h at a light intensity of 1400-1725 lux. The plants used for bioassays were grown in 10 cm diameter plastic pots until five to six true leaves were present, and the second and third leaves from the top of the plant (the highest fully expanded leaves) were used for all tests. All plants were watered as per necessary and fertilized with “Harvest More 20-20-20+TE”, a dry soluble fertilizer at a rate of 1 g/L water in seven-day intervals.

### Whitefly culture

*Bemisia tabaci* culture originated from our insectaries at the Key Laboratory of Applied Entomology, Northwest A&F University, Shaanxi, China, where it was previously cultured on tomato (*Solanum lycopersicum* *var.* ‘Florida Lanai’). Adult *B. tabaci* used in this study was identified by mitochondrial COI gene as biotype B [36]. Later, the whiteflies were cultured separately (stock rearing) on eggplant, tomato and cucumber, namely Bemisia-tomato, Bemisia-eggplant and Bemisia-cucumber, for 8-10 generations, but did not have a pepper population due to high mortality [15,29,37]. The whiteflies were cultured in large screen cages (65 × 65 × 65 cm) inside air conditioned insectaries with the environmental conditions of 25 ± 2°C, RH 65 ± 5%, and a photoperiod of 16L:8D h at a light intensity of 1400-1725 lux. Newly emerged adult whiteflies were used for bioassays and tests were done at the same environmental conditions.

### Host selection and oviposition of adults

Agar medium was prepared in 8.5 cm diameter sterilized clear plastic petri dishes. Leaf discs, 2 cm in diameter, were used to standardize the leaf area from each of the four plant species. Four leaf discs, one from each tested plant species, were placed randomly in a circle with equal distance among the leaf discs on the agar medium to keep the leaf discs fresh. The leaf discs were placed with the adaxial surface down on the agar and the abaxial surface up to examine feeding choice. Then the petri dishes with leaf discs were placed upside down to simulate leaves on the plants with the abaxial surface down. Twenty males and 20 females of adult whiteflies (10 adults/disc) were released into each petri dish. The movement of adults among leaf discs for their feeding preference and behavior was recorded continuously for 24 h using a digital CCD camera (LX-IR920Y, 1/3″ SONY 700TVL, Japan). Every test was initiated at 10:00 am each day. Video recording was aided with a 25-W lamp during the dark period. The recorded video was replayed continuously on a computer monitor to observe movement of whitefly adults for feeding and leaf disc selection. Then, number of whitefly eggs on each leaf disc was counted under a stereomicroscope. Eight replications were made with a completely randomized design.

### No choice oviposition

A leaf clip-on cage was used in this bioassay, while the cage was made according to Mouttet et al. [38] with some modifications. In brief, the size of the clip cage was 3.5 cm in diameter × 4 cm in height and about 5.25 g weight, and was made of a plastic cup where a metallic clip was used to hold the cage on abaxial leaf surface. There had a circular opening (2.5 cm in dimater) at the bottom of the cage which was covered with nylon mesh netting for ventilation, and a small hole (2-3 mm in diameter) was made at the side of cage to introduce adult whiteflies. The leaf clip-on cage was attached on the abaxial surface of a leaf of each of the four plant species. One mated female (24-48 h old) was introduced into each clip-on cage, and numbers of eggs laid on abaxial surface of the leaf were counted after three days of female introduction. Four plants of each species and two top fully expanded leaves were used. Each leaf was considered as one replication.

### Development and survivorship of whitefly

Four plants, one from each species were used, and two top fully expanded leaves were selected from each plant. Thirty couples of newly emerged adult whiteflies developed on each three colonies were confined in leaf-clip on cage onto the abaxial leaf surfaces of each of the four plant species. All whiteflies were removed from the cages 4 hours after adult introduction to get homogeneously aged eggs. Approximately 20-35 eggs were kept on each leaf which was considered as a replication of each treatment. Among them ten eggs were randomly selected and marked for developmental study (from egg to adult eclosion) while all eggs were monitored for survivorship. The immatures were exmined daily under a stereomicroscope until they either developed to adults or died.

### Data Analysis

First, we tested the normality of all data using the Shapiro-Wilk test with the null hypothesis that all data were normally distributed; and we found that the *P*-values in all data sets were greater than 0.05, and then we accepted our null hypothesis that our data were normally distributed [39]. In the feeding choice bioassays, the data of the number of adults moved on different leaf discs were analyzed through linear mixed model with repeated measurements of time where the time factor resulted in significant differences only on Bemisia-cucumber population. Therefore, further factorial ANOVA with time factor was carried out for the Bemisia-cucumber (PROC MIXED; SAS Version 9.2) [40]. The data of number of eggs in both the choice and no choice bioassays for oviposition, development duration and survival of immature bioassays were subjected to factorial ANOVA to test the effect of whitefly populations, host plants and their interactions as well. Means were separated using the least significant difference test (LSD) at *P* ≤ 0.05 when significant treatment effects were detected [40]. 

## Results

### Feeding and oviposition choice

**Figure 1:**
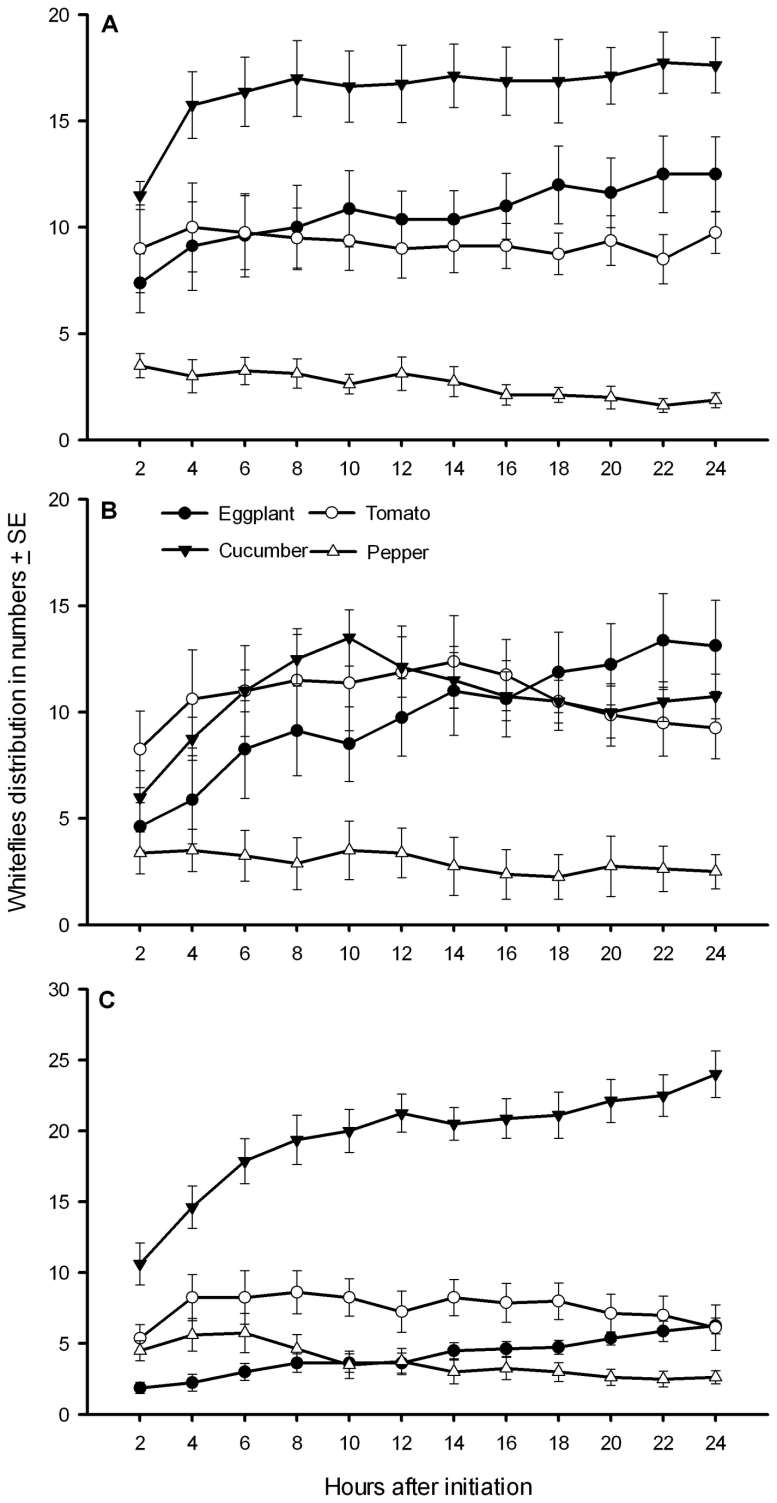
Numbers of *B. tabaci* adults (mean ± SE) on leaf discs of the four host plants at 2-h intervals for 24 h in a choice bioassay. A, B and C are denoted as Bemisia-eggplant, Bemisia-tomato, and Bemisia-cucumber, respectively.

**Figure 2:**
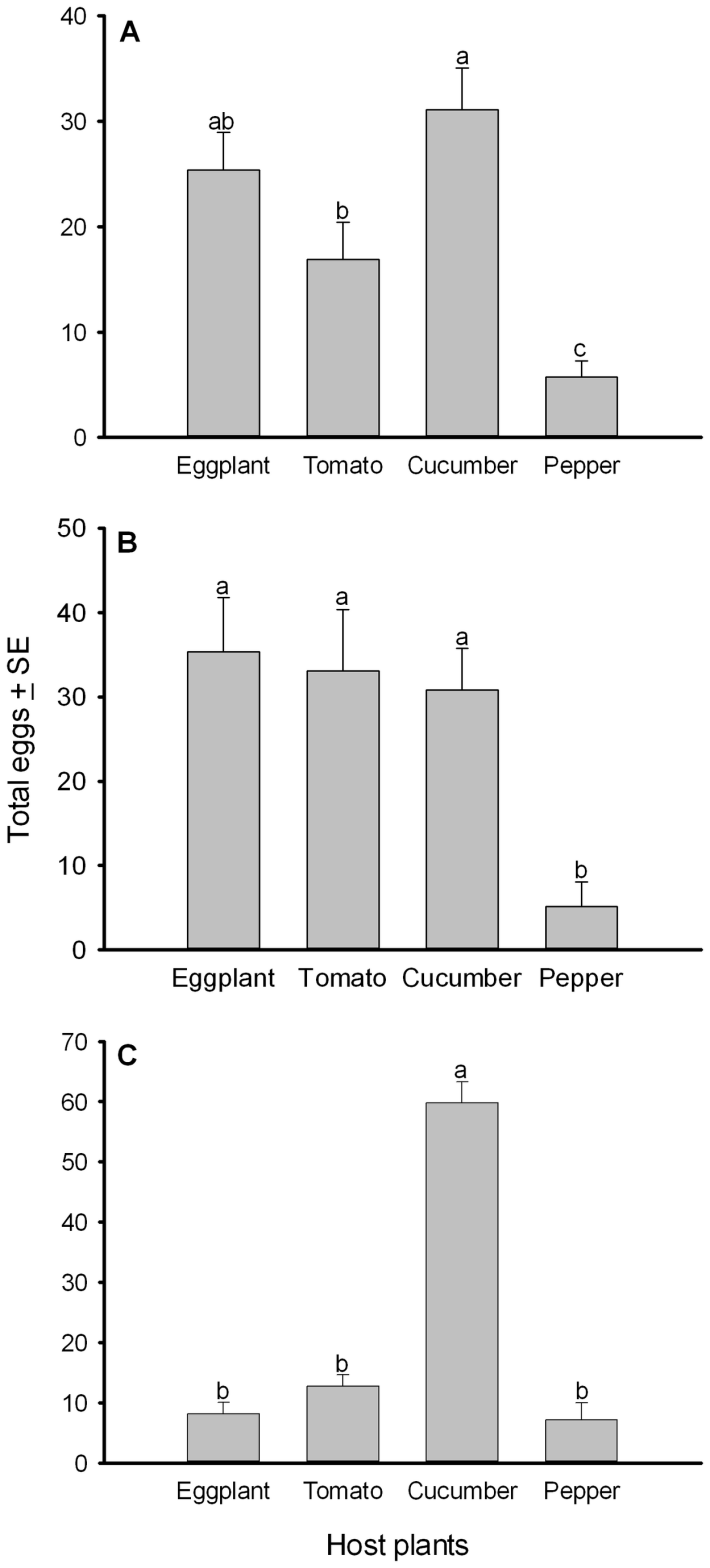
Number of *B. tabaci* eggs (mean ± SE) laid on leaf discs of the four host plants in a choice bioassay. A, B and C termed as total eggs laid by Bemisia-eggplant, Bemisia-tomato, and Bemisia-cucumber, respectively. Different letters above the error bars indicate significant difference at *P* ≤ 0.05, LSD.

Adult distribution dynamics in feeding choice of different whitefly populations on various host species are shown in Figure 1. The feeding choices of whitefly populations varied greatly (F_2,1008_ = 10.16, *P* < 0.001) to their natal host and other offered host plants. The Bemisia-eggplant showed significant differences for feeding among the plants (*F*_3,336_ = 199.60, *P* < 0.001) but not with time factor (*F*_11,336_ = 0.84, *P* = 0.605) and had no significant interactions (*F*_33,336_ = 0.67, *P* = 0.922) (Figure 1A). However, cucumber attracted the highest number of adults among the four host plants at 4-24 h after adult introduction and pepper attracted the least number, whereas eggplant and tomato showed intermediate attraction. In case of Bemisia-tomato, the adults attracted varied on different host plants (*F*_3,336_ = 65.71, *P* < 0.001), but not either with time factor (*F*_11,336_ = 1.87, *P* = 0.042) or their interactions (*F*_33,336_ = 1.05, *P* = 0.393) as shown in Figure 1B. Tomato had more whiteflies than other host plants 4 and 6 hours after adult introduction, and eggplant had more adults 18, 20, 22 and 24 hours after adult introduction, whereas pepper had the lowest number of adults all over the time. In contrast, the Bemisia-cucumber showed significant feeding preferences regarding plants (*F*_3,336_ = 486.16, *P* < 0.001), times (*F*_11,336_ = 3.63, *P* < 0.001), and their interactions (*F*_33,336_ = 3.15, *P* < 0.001) (Figure 1C). The greatest number of adults were found on cucumber at a 2-h interval for 24 h (2 h: *F*_3,28_ = 14.317, *P* < 0.001; 4 h: *F*_3,28_ = 16.568, *P* < 0.001; 6 h: *F*_3,28_ = 19.955, *P* < 0.001; 8 h: *F*_3,28_ = 30.815, *P* < 0.001; 10 h: *F*_3,28_ = 44.451, *P* < 0.001; 12 h: *F*_3,28_ = 53.157, *P* < 0.001; 14 h: *F*_3,28_ = 62.596, *P* < 0.001; 16 h: *F*_3,28_ = 54.335, *P* < 0.001; 18 h: *F*_3,28_ = 53.764, *P* < 0.001; 20 h: *F*_3,28_ = 64.939, *P* < 0.001; 22 h: *F*_3,28_ = 65.922, *P* < 0.001; 24 h: *F*_3,28_ = 64.297, *P* < 0.001) (Figure 1C). Eggplant had the least number of adults at 2-8 hours, and pepper had the least number of adults at 14-24 h. The oviposition in choice tests varied significantly among the populations (*F*_2,84_ = 3.74, *P* = 0.023), plants (*F*_3,84_ = 32.99, *P* < 0.001) and their interactions (*F*_6,84_ = 12.06, *P* < 0.001) (Figure 2.) For Bemisia-eggplant, the whiteflies oviposited the highest number of eggs (*F*_3,28_ = 11.322, *P* < 0.001) on cucumber and eggplant whereas the whiteflies oviposited the lowest number of eggs on pepper (Figure 2A). The females of Bemisia-tomato oviposited similar numbers of eggs on eggplant, tomato and cucumber, and less on pepper than on those three plants (*F*_3,28_ = 6.276, *P* < 0.002) (Figure 2B). The female adults of Bemisia-cucumber oviposited significantly more eggs on cucumber (*F*_3,28_ = 92.181, *P* < 0.001) among the four plants (Figure 2C). 

### No choice oviposition

The numbers of eggs laid in no choice oviposition bioassay significantly differed among whitefly populations (*F*_2,84_ = 6.85, *P* = 0.002), host plants (*F*_3,84_ = 39.30, *P* < 0.001), and their interactions (*F*_6,84_ = 5.81, *P* < 0.001) (Table 1). The female adults of Bemisia-eggplant laid the greatest number of eggs (*F*_3,28_ = 24.022; *P* < 0.001) on eggplant; whereas the females of Bemisia-tomato and Bemisia-cucumber laid the greatest number of eggs (*F*_3,28_ = 21.403; *P* < 0.001 and *F*_3,28_ = 9.330; *P* < 0.001, respectively) on cucumber. All *B. tabaci* populations deposited the least number of eggs on pepper.

**Table 1:** Number of eggs per female deposited by the three whitefly populations in no-choice bioassay, Bemisia-eggplant, Bemisia-tomato and Bemisia-cucumber, on different host plants in 3 days.

	Mean number of eggs (± SE)		
Host plants	Bemisia-eggplant	Bemisia-tomato	Bemisia-cucumber
Eggplant	34.8 ± 2.9 a	14.6 ± 1.9 b	19.8 ± 2.8 b
Tomato	20.5 ± 2.0 b	17.9 ± 1.2 b	16.4 ± 3.1 bc
Cucumber	22.4 ± 2.6 b	24.9 ± 3.3 a	29.1 ± 2.8 a
Pepper	6.9 ± 1.6 c	2.4 ± 0.7 c	9.1 ± 2.1 c

Means having the same letter in the same column were not significantly different at *P* ≤ 0.05, LSD

### Immature development duration

**Figure 3:**
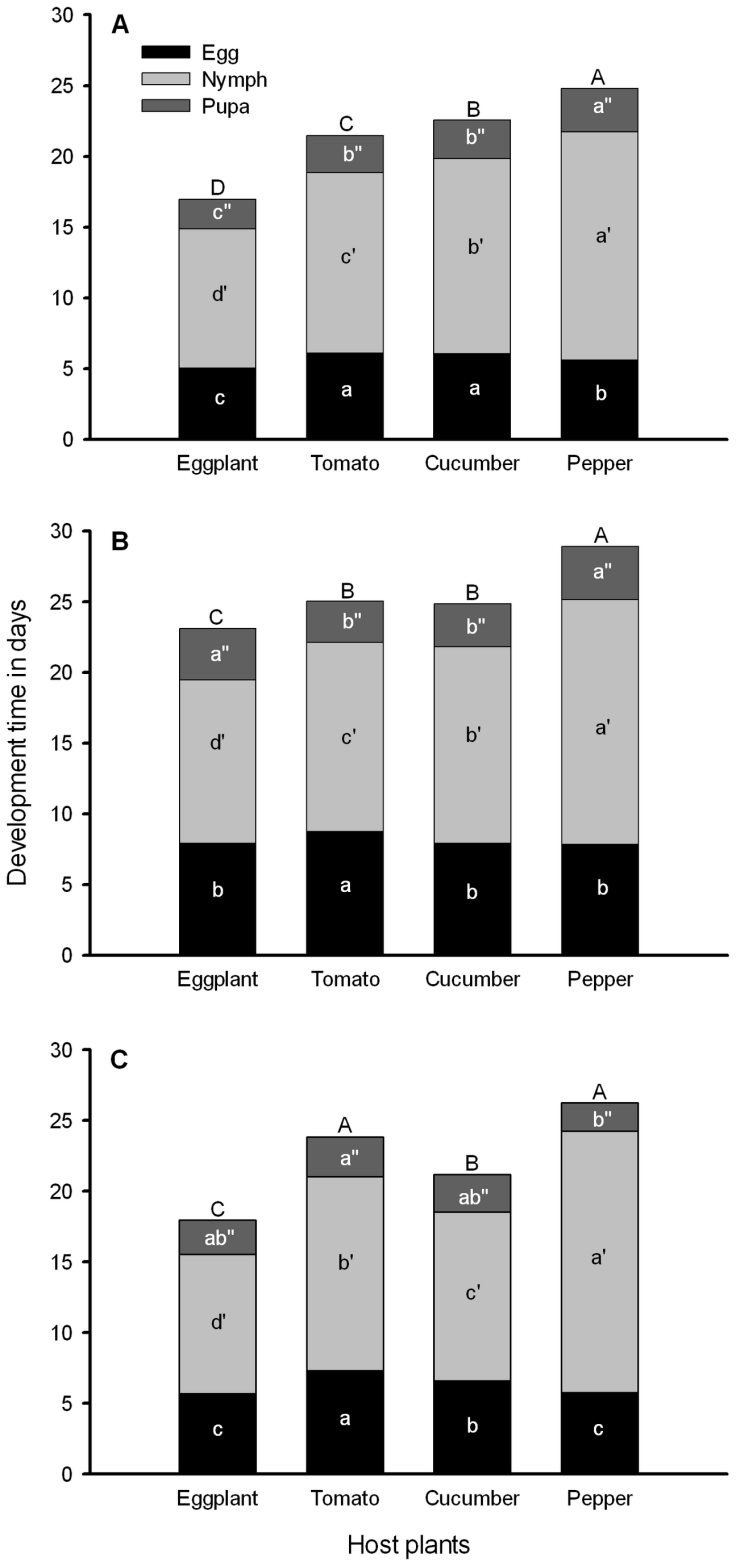
Development time (days ± SE) of *B. tabaci* immatures reared on four host plants. The bold A, B and C symbolized as immatures of Bemisia-eggplant, Bemisia-tomato, and Bemisia-cucumber, respectively. The regular A, B, C and D above the column symbolized for significant differences of overall development time among immatures on different host plants at *P* ≤ 0.05 (LSD). The letter on the columns a, b, c and d; a´, b´, c´ and d´; a", ab", b" and c" indicate significantly difference of the development time among of egg, larval and pupal stages, respectively, at *P* ≤ 0.05 (LSD).

Development durations from egg to adult emergence of all three populations of whiteflies varied greatly among the four plant hosts (*F*_3,747_ = 528.30, *P* < 0.001) and their interactions (*F*_6,747_ = 46.23, *P* < 0.001) (Figure 3).

#### Bemisia-eggplant

The incubation period of eggs was shorter on eggplant (*F*_3,316_ = 76.820; *P* < 0.001), longer on tomato and cucumber, and intermediate on pepper (Figure 3A). Development times of nymphs on each of the four plants differed (*F*_3,271_ = 194.393; *P* < 0.001), and the development times of the nymphal stages can be ranked from shortest to longest as: eggplant < tomato < cucumber < pepper. The development time of the pupal stage was shortest on eggplant (*F*_3,271_ = 49.039; *P* < 0.001) and the longest on pepper whereas no difference between cucumber and tomato, showed intermediate (Figure 3A). The overall development time of the immature stages varied greatly among the four plants (*F*_3,271_ = 252.857; *P* < 0.001), and can be ranked from shortest to longest as: eggplant < tomato < cucumber < pepper (Figure 3A).

#### Bemisia-tomato

The egg incubation period was longer on tomato than on other three hosts (Figure 3B). However, nymphal development time on each of the four plants differed (*F*_3,255_ = 262.075; *P* < 0.001), and the durations can be ranked from shortest to longest as: eggplant < tomato < cucumber < pepper (Figure 3B). The pupal development time was shortest on tomato and cucumber and the longest on eggplant and pepper (*F*_3,254_ = 28.435; *P* < 0.001). The overall durations of all immature stages differed among the four plants (*F*_3,254_ = 151.599; *P* < 0.001), the longest on pepper (28.3 ± 0.4, days ± SE), the shortest on eggplant (23.1 ± 0.1, days ± SE), and the intermediate on cucumber and tomato (Figure 3B). 

#### Bemisia-cucumber

The egg incubation periods differed among the four plants (*F*_3,306_ = 55.914; *P* < 0.001), the longest on tomato, followed by that on cucumber, and the shortest on eggplant and pepper (Figure 3C). The development times of the nymphal stages differed on the four plants (*F*_3,223_ = 137.390; *P* < 0.001). The durations can be ranked from shortest to longest as: eggplant < cucumber < tomato < pepper (Figure 3C). The overall development times of all immature stages were significantly different (*F*_3,222_ = 179.657; *P* < 0.001); the longest on pepper and tomato, and the shortest on eggplant, and intermediate on cucumber (Figure 3C).

### Immature survivorship

**Figure 4:**
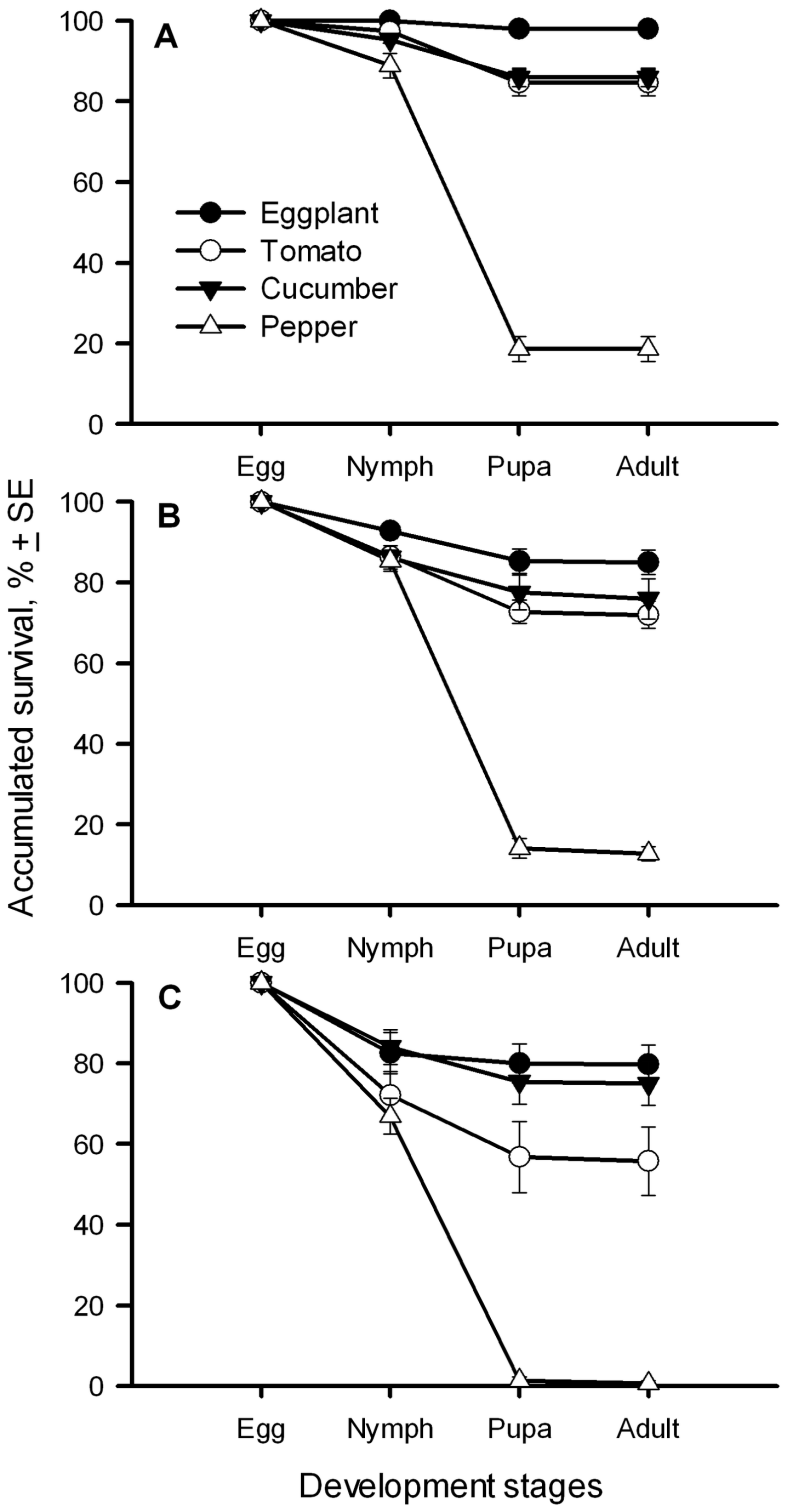
Survivorship (% ± SE) of *B. tabaci* immatures reared on four host plants. A, B and C represented for the immatures of Bemisia-eggplant, Bemisia-tomato, and Bemisia-cucumber, respectively.

The overall immature survival rates varied significantly among the whitefly populations (*F*_2,84_ = 21.92, *P* < 0.001) and among the plant species (*F*_3,84_ = 220.51, *P* < 0.001) (Figure 4).

#### Bemisia-eggplant

The survival rate of eggs varied among the four hosts (*F*_3,28_ = 7.004; *P* < 0.001) (Figure 4A). All eggs developed to nymphs on eggplant (100.0 ± 0.0%), more than 95% on tomato and cucumber, and almost 89% on pepper. The survival of nymphs varied greatly among the plants (*F*_3,28_ = 189.176; *P* < 0.001). The greatest survival was found on eggplant (98.0% ± 0.9) as natal host and the least number survived on pepper (20.5% ± 3.1) and were intermediate on tomato and cucumber (Figure 4A). All pupae successfully developed to adults on all plants. The overall survival rates of immatures varied among the plant species (*F*_3,28_ = 198.09, *P* < 0.001) and can be ranked from the highest to the lowest as: eggplant > tomato and cucumber > pepper.

#### Bemisia-tomato

The survival of the eggs was similar on the four host plants. Although nymph survival rates differed significantly on the four plant hosts (*F*_3,28_ = 140.854; *P* < 0.001) (Figure 4B), only that on pepper was lower. Almost all pupae successfully developed to adults on all plants. The overall survival rates of Bemisia-tomato immatures also differed on various plant species (*F*_3,28_ = 88.11, *P* < 0.001) though there had no differences among eggplant, tomato and cucumber.

#### Bemisia-cucumber

There was no significant difference in egg survivorship among the four plant hosts (*F*_3,28_ = 2.768; *P* < 0.060) (Figure 4C). However, the survival of the nymphs of the Bemisia-cucumber varied significantly among the four plant hosts (*F*_3,28_ = 134.403; *P* < 0.001) (Figure 4C). The nymphal survival rates were highest on eggplant and cucumber, and lowest on pepper, and intermediate on tomato. Similar to other populations, almost all pupae successfully developed to adults on all plants. The survival rate of all immature stages (egg to adult) varied among the plant species (*F*_3,28_ = 42.57, *P* < 0.001) and can be ranked from the highest to the lowest as: eggplant and cucumber > tomato > pepper. 

## Discussion

Our data showed that the effects of previous host plant experiences of *B. tabaci* varied, and host plants played a more important role than previous experience. We found that the Bemisia-eggplant and Bemisia-cucumber preferred cucumber for feeding. Lei et al. [41] also stated that cucumber was more acceptable for probing and feeding. Our results show that the most preferred host plants for feeding might not be the best host for immature development and survival. For instance, *B. tabaci* immatures on eggplant had 80-98% survival and the shortest development duration, which were better than on any of host plants tested in this study. However, the whitefly populations with experience on eggplant preferred cucumber (eggplant ranked second) for feeding. In contrast, Bemisia-cucumber preferred cucumber much better than any other host plants. This phenomenon has been thought to reflect the feeding experience recognition, i.e. whitefly population chose their natal host for feeding, and this is in agreement with the results of Lee et al. [42,43]. The preference of tomato for feeding by all whitefly populations ranked third, and our data of immature development and survival on tomato support that tomato is a poorer host plant than cucumber and eggplant. Perhaps tomato was a less attractive host for adult whiteflies in the presence of more preferred hosts. However, Bemisia-tomato preferred tomato at earlier hours during feeding choice which reflects the effect of feeding experience. This might be due to long term association with specific host plant that mediates the behavior of whitefly. 

Pepper is generally not a good host for B biotype of *B. tabaci* [15,29,37]. Our data showed that all populations of *B. tabaci* did not prefer pepper plant in the choice test, and the all populations on pepper gradually decreased. Through the video recording, we found that at earlier hours the adults landed on pepper, but after labial contact or feeding, the adults relocated to other hosts. This result suggests that further investigations on feeding behavior are needed to address this phenomenon properly that could be done by the electrical penetration graph (EPG) technique [41]. In general, the host selection process of whiteflies is primarily by labial contact with host surface [41]. Our results suggest that whitefly populations affect on feeding choice but the host plant had stronger role than previous experience. This observation supports with the previous finding [41], which referred that the host plants had a stronger influence in feeding activities of the whiteflies than the whitefly strains. 

In choice test, Bemisia-eggplant and Bemisia-cucumber deposited more eggs on cucumber than on other host plants, reflecting that host plant is a major factor for the manipulation of whitefly oviposition. The number of eggs laid on eggplant by Bemisia-eggplant did not differ from that on cucumber. On the other hand, number of eggs of Bemisia-cucumber on cucumber was much higher than on other host plants. These results demonstrate that previous host experience by whitefly populations can influence subsequent host plant selection. The Bemisia-tomato oviposited similar number of eggs on eggplant, tomato and cucumber though finally the adults preferred tomato plant thirdly among four plants. We observed that at earlier hours more adults of the Bemisia-tomato were found on tomato, assuming more adults laid more eggs; subsequently some of the adults relocated to other plants, and eventually there were similar numbers of eggs on eggplant, cucumber and tomato. However, all populations laid the lowest number of eggs on pepper. The number of eggs per adult was not significantly different among the plants for all populations of *B. tabaci*. This is due to the fact that adults landed on plants and laid eggs, but after contact the labial palps on the leaf surfaces they relocated and settled on their preferred hosts. This phenomenon has been assumed that adults laid eggs first, subsequently settled on leaves for feeding. Therefore, number of eggs per adult may not accurately reflect the host suitability of adults, when they have choice for feeding and settling. In the no choice test, Bemisia-eggplant and Bemisia-cucumber deposited the greatest number of eggs on their natal host plants which were in agreement with the result of Brown et al. [44]. This result also reflects the fact of feeding experience. In contrast, Bemisia-tomato laid more eggs on cucumber. These results showed that all *B. tabaci* populations did not prefer pepper. This might be due to the fact that pepper foliage was glabrous, whereas all others three host plants were pubescent because *B. tabaci* preferred the plants with pubescent leaves to the plants with glabrous leaves for feeding and oviposition. These results were similar to those reported by Gruenhagen and Perring [22], Mansaray and Sundufu [32], and Khan et al. [45]. 

The result of our study showed that developmental time from egg to adult eclosion of the Bemisia-eggplant, Bemisia-tomato and Bemisia-cucumber varied greatly when reared separately on eggplant, tomato, cucumber, and pepper. The incubation periods of all populations were always longer on tomato than on the other three hosts, indicating that tomato is a poorer host for eggs development than others. These results suggest that eggplant was better for development of *B. tabaci* immatures, and pepper was a poorer host plant, where tomato and cucumber were marginal, reflecting that host plant is a main factor that influences the development of immature whiteflies. However, tomato ranked second for development of the Bemisia-tomato, and cucumber ranked second for Bemisia-cucumber. These results clearly reflect the effect of whitefly populations of previous experience. 

Nymphal survival was more significant among the host plants in all whitefly populations. The first and second instars on pepper plant had the lowest survival rate. The survival of the red-eyed nymphs or pupae was highest among other immature stages. The overall survival of all populations from egg to adult eclosion was highest on eggplant (80-98%) and lowest on pepper (0.6-20%). Similar results have been reported in previous studies [15,29,34,37]. Again, this result confirmed that host plant had the foremost effect on the survival of whitefly immatures. The immature survival of the Bemisia-cucumber on other three hosts (eggplant, tomato and pepper) was lower than the Bemisia-eggplant and Bemisia-tomato immature on those hosts. This might be due to the distant phylogenetic relation among cucumber and other three hosts, which supports the result reported by Carabali et al. [46].

In this study, Bemisia-eggplant performed better for their immature development as the shortest development duration and the highest survival on either natal or offered host plant.This result supports with the result of Enkegaard [47] who stated that the poinsettia strain performed better than the tobacco strain on poinsettia. Accordingly, adult feeding and egg laying preference and immature performance of *B. tabaci* mainly manipulated by plant features and partially by whitefly populations, supported by the results of Thomas [48] and Jiao [35]. The plant features include the nutrition of phloem sap, presence of secondary metabolites, and leaf surface characteristics [49,50]. 

In summary, the findings of our study can be stated as: firstly, feeding choice and oviposition of adults, and development and survival of immatures were mediated by both whitefly populations and host plants, though host plants had stronger effects. Secondly, whiteflies from different host plants performed differently on different host plants; *B. tabaci* adults may land on the plants that are not preferred and laid eggs first, and then relocate and settled on the plants they preferred. Therefore, numbers of adults that land on the plants may not be used to interpret for host suitability of immatures of *B. tabaci*. Thirdly, cucumber may be used as a trap crop for management of *B. tabaci*. Our findings will be useful for further understanding the population dynamics, dispersal and viral transmission in a landscape or large cropping system where *B. tabaci* on different crops spread around different crop systems. 
